# Microbubble-mediated delivery of human adenoviruses does not elicit innate and adaptive immunity response in an immunocompetent mouse model of prostate cancer

**DOI:** 10.1186/s12967-019-1771-0

**Published:** 2019-01-11

**Authors:** Flavia De Carlo, Litty Thomas, Bell Brooke, Elliot T. Varney, Rounak Nande, Olivia Boskovic, Gailen D. Marshall, Pier Paolo Claudio, Candace M. Howard

**Affiliations:** 1 Department of BioMolecular Sciences, University of Mississippi, University, MS USA; 2 National Center for Natural Products Research, University of Mississippi, University, MS USA; 3 Department of Radiation Oncology, Medical Center Cancer Institute, Jackson, MS USA; 4 Department of Radiology, University of Mississippi Medical Center, Jackson, MS 39126 USA; 5 Department of Biochemistry and Microbiology, Joan C. Edwards School of Medicine, Marshall University, Huntington, WV USA; 6 Division of Clinical Immunology and Allergy, Department of Medicine, University of Mississippi Medical Center, Jackson, MS USA; 7 Department of BioMolecular Sciences, Department of Radiation Oncology, University of Mississippi, Jackson, MS 39126 USA

**Keywords:** Microbubbles, Gene transfer, Prostate cancer, Innate immunity, Adaptive immunity

## Abstract

**Background:**

Gene transfer to malignant sites using human adenoviruses (hAds) has been limited because of their immunogenic nature and host specificity. Murine cells often lack some of the receptors needed for hAds attachment, thus murine cells are generally non-permissive for human adenoviral infection and replication, which limits translational studies.

**Methods:**

We have developed a gene transfer method that uses a combination of lipid-encapsulated perfluorocarbon microbubbles and ultrasound to protect and deliver hAds to a target tissue, bypassing the requirement of specific receptors.

**Results:**

In an in vitro model, we showed that murine TRAMP-C2 and human DU145 prostate cancer cells display a comparable expression pattern of receptors involved in hAds adhesion and internalization. We also demonstrated that murine and human cells showed a dose-dependent increase in the percentage of cells transduced by hAd-GFP (green fluorescent protein) after 24 h and that GFP transgene was efficiently expressed at 48 and 72 h post-transduction. To assess if our image-guided delivery system could effectively protect the hAds from the immune system in vivo, we injected healthy immunocompetent mice (C57BL/6) or mice bearing a syngeneic prostate tumor (TRAMP-C2) with hAd-GFP/MB complexes. Notably, we did not observe activation of innate (TNF-α and IL-6 cytokines), or adaptive immune response (neutralizing antibodies, INF-γ+ CD8^+^ T cells).

**Conclusions:**

This study brings us a step closer to demonstrating the feasibility of murine cancer models to investigate the clinical translation of image guided site-specific adenoviral gene therapy mediated by ultrasound-targeted microbubble destruction.

**Electronic supplementary material:**

The online version of this article (10.1186/s12967-019-1771-0) contains supplementary material, which is available to authorized users.

## Background

Human adenoviruses (hAds) are highly effective gene transfer agents that can introduce different types of genetic materials into cancer cells, including tumor suppressor genes [1]. Taxonomically grouped into the *Adenoviridae* family, adenoviruses are known to infect a wide variety of species [2]. Human adenoviruses are non-enveloped, icosahedral viruses, approximately 90 nm in diameter with a fiber complex known as knob domain that binds to the Coxsackie and Adenovirus Receptor (CAR), thus mediating cell tropism [3–5]. Interaction of adenoviral penton proteins with surface integrins such as α_V_β_3_ and α_V_β_5_ assists in the internalization of the virus; however, horizontal gene transfer of adenoviruses is often difficult due to the strict host specificity demonstrated by the viruses [6]. Generally, murine cells lack some of the receptors needed for hAd infection, such as CAR, thus making them generally non-permissive for hAd infection and replication. However, a very low level of hAd infection and replication has been described in some mouse cells [7, 8].

Human adenoviruses serotypes 2 and 5, classified under species type C, have shown promising results in treating locally advanced cancers, but these adenoviruses are highly immunogenic triggering both innate and adaptive immune responses [3, 4]. The innate immune response is elicited in the professional antigen presenting cells (APC) by hAds through the myeloid differentiating factor 88 (MyD88)/Toll-like receptor (TLR)-9 dependent or independent pathways resulting in the up-regulation of type I interferons (IFNs) and inflammatory cytokines such as TNF-α, IL-6 and IL-12 [9, 10]. Complement, another key component of the innate immunity, has an important role in the opsonization and clearance of adenoviruses. Complement activation can occur via direct binding of adenovirus with C3-derived fragments or through neutralizing antibodies produced after a previous immunization [11]. Viral exposure leads to innate and adaptive immune system interaction resulting in the differentiation of B cells into antibody-secreting plasma cells and the differentiation of T cells to cytotoxic T lymphocytes (CTLs). Anti-adenovirus 5 serotype antibodies have been found to target several components of the capsid, including hexons and fiber knobs, after both vaccination and natural infection to mediate virus neutralization [2, 12]. Specifically, the humoral response causes a reduction in the viral load hampering the systemic re-administration of adenovirus in protocols of gene therapy [11]. While neutralizing antibodies (NAbs) prevent re-administration of the vector, the antigen-specific T cell response, mediated by CTLs, limits the duration of transgene expression and eliminates transduced cells. Therefore, the success of long-term gene therapy is dependent on the ability to avoid the induction of immune responses against both vector and the transgene product [13].

When adenoviruses are directly administrated via the circulatory system, 85–98% of the viral dose is accumulated in the liver within 30 min, and the remaining is found in lung, kidney, and spleen resulting in off-target interactions and systemic toxicity [2, 14]. Moreover, CAR is present in most human cell types that contribute to off-target transduction or non-specific interactions [2, 6].

In humans, in the absence of pre-exiting immunity (rare in humans for Ad5 based vectors), the virus may bind blood clotting factors [15], IgM [16] and/or erythrocytes [17]. All this leads to rapid active RES (Kupffer cell) mediated capture and rapid clearance from the blood [18]. The sinusoidal endothelial spaces in the human liver measure 105 nm [19] i.e. smaller than the diameter of a virus with intact fibre domains, providing minimal hepatocyte access and infection, hence the minimal liver toxicity seen in the clinics compared to mice [20]. This is not recapitulated in mice as they do not have CAR on their erythrocytes and have a liver sinusoidal endothelial gap size of 130–160 nm depending on strain. Hence, RES capture but also very high levels of liver infection and toxicity are seen in mice.

In humans with pre-existing immunity neutralization and RES capture may be even more effective. Unfortunately, this is not recapitulated in research mice because they do not have pre-existing immunity, therefore in our studies we tried to circumvent this by injecting the mice two times with the hAds to simulate pre-existing immunity.

The aforementioned limitations have restricted the use of hAds for gene therapy to direct intratumoral (IT) or organ injection [21]. To overcome these limitations, we developed a systemic site-specific delivery system where ultrasound (US) contrast agents, here referred as microbubbles (MBs), are used as delivery vehicles. These hAds, loaded inside shells of acoustically active, lyophilized, lipid-encapsulated, perfluorocarbon filled MBs, are released when MBs are destroyed by US at the tumor site. These bubbles range between 2.5 and 4.5 µm, and after injection into the bloodstream, they can re-circulate through the vascular system numerous times for several minutes with minimal accumulation and interaction [21–23]. Their small dimension prevents entrapment within the pulmonary capillary bed (~ 5 to 8 µm), yet still enable proper protection of the viral vectors, such as hAds, from the environment [21]. MBs protect the viral payload from detection and rapid degradation by the hosts’ immune system allowing for an intravenous (IV) inoculation rather than intratumoral (IT) injection [21, 24]. US breaks open the MB/hAds complexes by inducing cavitation, allowing the hAds to transfer their transgene to the sonoporated region. Cavitation of the MBs causes small shockwaves, which increase cell permeability by forming temporary micropores on the cell surface, bypassing the receptor-mediated dependence of hAds cellular transduction.

In the recent past, we have successfully utilized this MB gene transfer system to selectively transfer both expression markers and therapeutic genes into tumors in immune deficient mice [21, 25–27]. In this study, we compared the transduction efficiency of hAd-GFP and GFP expression in the mouse prostate cancer cell line (TRAMP-C2, C57BL/6 background) and the human DU145 prostate cancer cell line. Additionally, using healthy immunocompetent C57BL/6 mice or mice bearing a syngeneic TRAMP-C2 prostate tumor, we evaluated the capability of ultrasound contrast agents to protect systemically-delivered adenoviral vectors from the innate and adaptive immune system using an in vivo model, and the contrast agent’s ability to prevent off-target distribution utilizing ultrasound-targeted microbubble destruction.

## Materials and methods

### Cell lines

The DU145 (human prostate adenocarcinoma, radio-resistant, p53 deficient, derived from a brain metastasis), TRAMP-C2 (prostate adenocarcinoma, radio-resistant, wild-type p53, derived from 32-week old TRAMP mice) and human kidney embryonic HEK-293 cell lines were obtained from the American Type Culture Collection (ATCC, Rockville, MD). DU145 cells were grown in RPMI-1640 (Hyclone, Waltham, MA) supplemented with 10% fetal bovine serum (FBS) (Hyclone), and 100-units/ml penicillin supplemented with 1 mg/ml streptomycin (Hyclone). TRAMP-C2 cells were grown in Dulbecco’s modified Eagle’s medium (Hyclone) supplemented with 5% FBS (Hyclone), 5% Nu-Serum IV (Corning, Corning, NY), 5 μg/ml bovine insulin (Sigma Aldrich, St. Louis, MO), 10 nM dehydroisoandrosterone 90% (Sigma Aldrich), and 100 units/ml penicillin supplemented with 1 mg/ml streptomycin (Hyclone). The HEK-293 cells were grown in Dulbecco’s modified Eagle’s medium (Hyclone) supplemented with 10% FBS (Hyclone). All cells were grown at 37 °C, in a 5% CO_2_ in 95% atmosphere incubator.

### Adenoviral production

Human adenovirus serotype 5 E1/E3 deleted, which expresses the GFP gene under the strong cytomegalovirus (CMV) constitutive promoter, was generated using the Ad Easy system (Agilent Technologies, Carlsbad, CA) then amplified and purified with the BD Adeno-X virus purification kit (BD Biosciences, Mountain View, CA) following manufacturer’s directions. Viral titers were determined by Tissue Culture Infectious Dose 50 (TCID_50_) and the titer was adjusted to 1 × 10^11^ plaque-forming units (PFU)/ml as described. Each viral stock was propagated and purified from infected HEK-293 cells, as previously published [21, 25, 27–29]. HEK-293 cells were harvested 48 h after infection, pelleted and suspended in medium. Cells were lysed by a three-freeze/thaw cycle method and cell debris were removed by centrifugation. Viruses were purified by chromatography followed by dialysis and stored at − 80 °C.

### Human adenovirus attachment receptors analysis

DU145 and TRAMP-C2 cells were analyzed for the expression of hAd attachment receptors: CAR (coxsackie adenovirus receptor), α_V_β_5_ and α_V_β_3_ integrins. Single cells suspension was obtained and cells were labeled with the following antibodies in cold FACS buffer (PBS 1× + EDTA 2 mM + FBS 0.5%): Rabbit anti-human CAR Monoclonal antibody FITC-conjugated (10799-R271-F, Sino Biologicals Inc, Beijing, China), Rabbit Anti-Integrin α_V_ + β_5_ Polyclonal Antibody Alexa Fluor^®^ 647 Conjugated (bs-1356R-A647, Bioss, Woburn, MA) and Rabbit Anti-Integrin α_V_ + β_3_ (CD51 + CD61) Polyclonal Antibody Alexa Fluor^®^ 488 Conjugated (bs-1310R-A488, Bioss). Rabbit Isotype control antibodies were used for background normalization. Cells were incubated for 30 min at 4 °C in the dark, then washed twice in FACS buffer. Cells were stained with 2 µg/ml of propidium iodide for dead cell exclusion. Samples were acquired with a BD Accuri C6 Flow Cytometer (BD Biosciences, San Jose, CA) and data analyzed by the CFlow Plus Analysis Software (BD Biosciences).

### Transduction efficiency

Adenoviral transduction efficiency was evaluated 24 h post-infection of mouse TRAMP-C2 and human DU145 cell lines with 10, 25, 50 MOI of hAd-GFP, using DMEM media or RPMI-1640 media with 2% heat-inactivated FBS (Hyclone), respectively. A qualitative analysis of the transduction efficiency was performed acquiring images of hAd-GFP infected cells by fluorescence microscopy using an inverted Olympus IX70 microscope (Olympus America, Inc. Melville, NY). Cells were counterstained with Hoechst 33342 (Molecular Probes, Eugene, OR). Additionally, the percentage of cells positive to GFP was measured 24-h post-infection of mouse TRAMP-C2 and human DU145 cell lines with 10, 25, 50 MOI of hAd-GFP by flow cytometry. Propidium iodide labeling was used to exclude dead cells.

### GFP gene expression

Transgene expression was assessed in TRAMP-C2 and human DU145 cell lines at 24, 48, and 72 h after infection with 10 MOI of hAds, using DMEM or RPMI-1640 media with 2% heat-inactivated FBS (Hyclone), respectively. GFP median fluorescence intensity was measured by flow cytometry. Propidium iodide staining was used to exclude dead cells.

### Preparation of microbubbles

Targeson, (Targeson, Inc. San Diego, CA) uniquely-constructed ultrasound contrast agents (perfluorocarbon microbubbles, encapsulated by a lipid monolayer and polyethylene glycol stabilizer), were prepared following manufacturer’s instructions. MBs were reconstituted in the presence or absence of 1 ml of 1 × 10^11^ plaque-forming units/ml of Ads and unenclosed, surface-associated Ads were inactivated, as previously described [21, 25, 27].

Briefly, unenclosed and free adenoviruses were inactivated by incubating 1 volume of microbubbles formed in the presence of Ad-GFP with 10 volumes of a solution containing 60 mg/ml of human complement (Sigma Aldrich, Saint Louis, MS) for 30 min at 37 °C. Microbubbles were then washed with 10 ml of phosphate buffer saline solution (PBS). The milky white suspension floating on the top of PBS was then collected and used in the in vitro and in vivo experiments. We delivered 10^9^ PFU Ad-GFP/mouse using the MBs/US system, and the titer was comparable to the one injected in the control mice (IV and IT injections) (see Additional file 1).

### In vivo ultrasound-targeted microbubble destruction

Animal studies were performed in accordance with National Institutes of Health recommendations and the approval of the Institutional Animal Care and Use Committee. Animal care and humane use and treatment of mice were in strict compliance with (i) institutional guidelines, (ii) the Guide for the Care and Use of Laboratory Animals (National Academy of Sciences, Washington, DC, 1996), and (iii) the Association for Assessment and Accreditation of Laboratory Animal Care International (Rockville, MD, 1997). All the animals used in these studies were 8–11 week-old male C57BL/6 (H2^b^) immunocompetent mice (Jackson Laboratories, Bar Harbor, ME). Two in vivo experiments were performed using a total of 30 mice divided into groups containing 3–6 mice for each experiment. The first experiment was performed in healthy C57BL/6 mice while the second utilized C57BL/6 bearing a syngeneic TRAMP-C2 tumor. To establish syngeneic tumor grafts, the mice were injected in the right flank with TRAMP-C2 prostate adenocarcinoma cell lines (5 × 10^6^ cancer cells) using a 20-gauge needle. Treatment was started when the tumor reached 50–100 mm^3^ of volume. During the experimental procedure, the mice were sedated using an IMPAQ6 anesthesia apparatus (VetEquip, Pleasanton, CA) saturated with 3–5% isofluorane and 10–15% oxygen with the aid of a precision vaporizer (VetEquip), and placed on a warming mat set at 37 °C. Treatments were delivered intravenously (IV) or intratumorally (IT) in a volume of 100 μl using a 30-gauge needle. US exposure was performed with a Micro-Maxx SonoSite ultrasound machine (SonoSite) equipped with the transducer L25 set at 0.7 Mechanical Index (MI), 1.8 MPa for 10 min [21, 25, 27].

### TNF-α and IL-6 quantification

Two hours after the first IV or IT injection, mice were deeply sedated and 100 μl of blood was collected by puncture of the mandibular vein using a Goldenrod Animal Lancet (Braintree Scientific, Inc., Braintree, MA). Mouse serum TNF-α and IL-6 levels were analyzed using Quantikine HS Mouse TNF-alpha (R&D System, Minneapolis, MN) and Quantikine Mouse IL-6 and (R&D System) Immunoassay solid-phase ELISAs, following manufacturer’s directions.

### Anti-adenovirus antibodies detection

At the experimental endpoints, mice were deeply anesthetized, and blood was collected from the heart. Afterwards, the mice were sacrificed by CO_2_ gas and cervical dislocation.

ELISA plates were coated overnight at 4 °C with 5 × 10^6^ vp/well of Ad-GFP. Plates were blocked for 2 h at room temperature with 3% BSA/PBS. Mice serum was heat inactivated at 56 °C for 30 min, diluted 1:3000 in 1%BSA/0.05%Tween20/PBS, added to the wells in triplicate and incubated for 2 h at room temperature. Reactive antibodies were detected using a secondary antibody sheep anti-mouse IgG HRP-conjugated (NA931, GE Healthcare, Chicago, IL). SureBlue TMB Peroxidase Substrate (KPL, Gaithersburg, MD) was added to each well and color development was assessed at 650 nm using a microplate reader.

### INF-γ ELISPOT

Immediately following mice euthanasia, spleens were collected and processed. Red blood cells were removed using a Red Blood Cells Lysis buffer (Affymetrix, Santa Clara, CA). Splenocytes were suspended at 2 × 10^6^ cells/ml in AIM V medium containing l-glutamine, streptomycin sulfate 50 µg/ml, and gentamicin sulfate 10 µg/ml, and supplemented with 50 µM 2-mercaptoethanol. Two hundred-thousand cells/well were stimulated with 2 µg/ml of DNA-binding protein peptide, corresponding to DBP_418–426_ (FASLNAEDL, H-2D^b^ restricted peptide, New England Peptide, Gardner, MA) [30] for 24 h. Stimulation of splenocytes with 0.05 µg/ml of anti-mouse CD3 was used as positive control (552774, BD Biosciences, Franklin Lakes, NJ). Splenocytes were subjected to ELISpot assay using the INF-γ ELISpot PLUS kit (Mabtech, Nacka Strand, Sweden) following manufacturer’s directions. Spots, corresponding to INF-γ secreting cells, were counted using a Zeiss ELISpot reader system (service provided by ZellNet, Inc. Fort Lee, NJ).

### Statistical analysis

Statistical analysis was performed using GraphPad Prism 6 statistical software (Graphpad, Inc., La Jolla, CA). One and two-way analysis of variance (ANOVA) with Tukey or Bonferroni multiple comparisons post-test was used to determine the statistical significance of the differences between experimental groups. Multiple t-test was used for the ELISpot analysis. p-values of less than 0.05 were considered statistically significant.

## Results

### TRAMP-C2 and DU145 cells express hAd receptors integrins αVβ5, αVβ3, and CAR

**Fig. 1:**
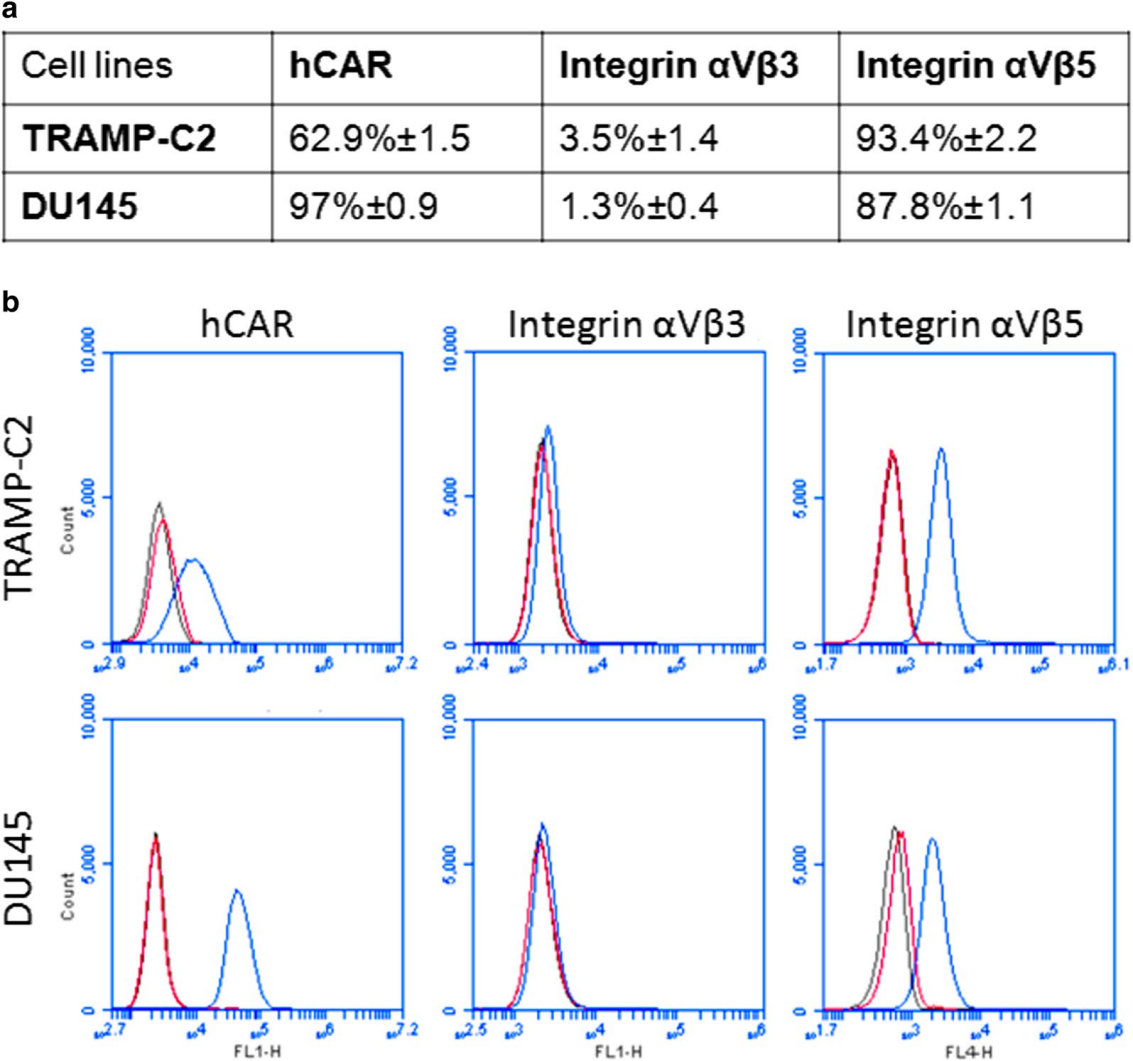
Human adenoviral receptors expression in TRAMP-C2 and DU145. **a** TRAMP-C2 and DU145 cells were labeled with Rabbit Anti-human Coxsackie adenovirus receptor FITC conjugated (hCAR), Rabbit Anti-Integrin α_V_ + β_5_ Alexa Fluor^®^ 647 Conjugated and Rabbit Anti-Integrin α_V_ + β_3_ Alexa Fluor^®^ 488 Conjugated. Percentages are represented as mean ± SEM of three biological repetitions. **b** Representative flow cytometer histograms showing the percentage of cells that are expressing human adenovirus receptors. Black: negative control, Red: isotype control, Blue: specific antibody

The expression profile of surface proteins known to be responsible for the specific attachment of hAd5 to human cells was evaluated by flow cytometry (Fig. 1a, b). Both human DU145 and mouse TRAMP-C2 prostate cancer cells were positive to the expression of integrin α_V_β_5_ and α_V_β_3_. The percentage of cells expressing human Coxsackie and Adenovirus Receptor (CAR), as expected, was higher in the human cell line DU145 when compared to the mouse TRAMP C2 cells (97 ± 0.9% vs. 62.9 ± 1.5%).

### TRAMP-C2 and DU145 cells are transduced by hAd-GFP

**Fig. 2:**
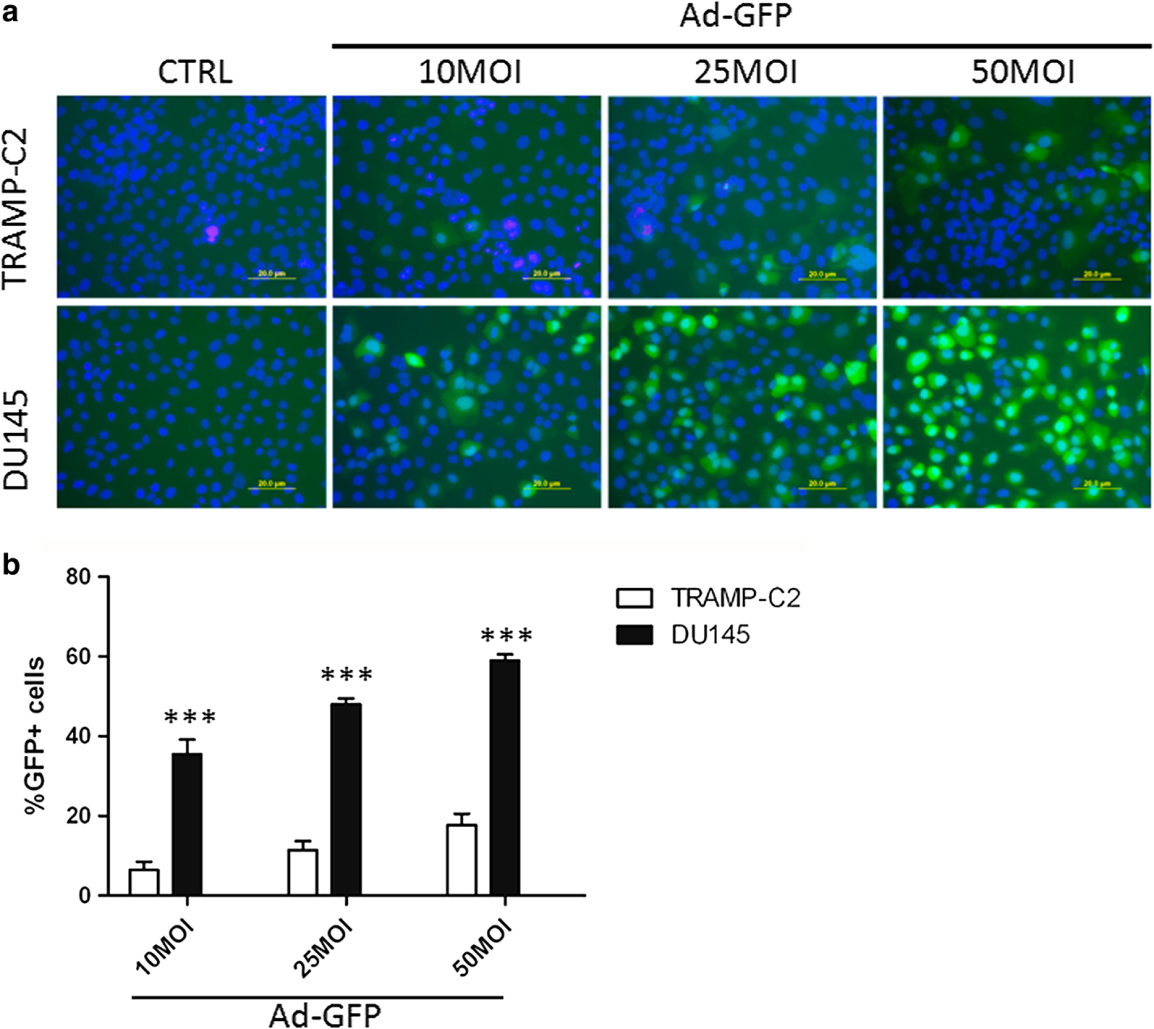
Human adenoviral transduction efficiency in TRAMP-C2 and DU145. **a** TRAMP-C2 and DU145 cells were transduced with 10, 25, 50 MOI of hAd-GFP for 24 h and images were acquired by fluorescence microscopy. Nuclei were counterstained with Hoechst 3334. **b** TRAMP-C2 and DU145 cells were transduced with 10, 25, 50 MOI of hAd-GFP for 24 h and the percentage of cells transduced were determined by flow cytometry. Data are representative of three biological repeats, analyzed by two way ANOVA, Bonferroni’s post-test ***p < 0.001

Despite the high host specificity of adenovirus, we wanted to define if murine cells could be infected by a hAd. The transduction efficiency of murine TRAMP-C2 and human DU145 prostate cancer cell lines by hAd-GFP was assessed 24 h post infection with 10, 25, and 50 MOI (multiplicity of infection) of hAd-GFP. Using fluorescence microscopy, we observed a dose-dependent increase of GFP positive cells in both TRAMP-C2 and DU145 cells (Fig. 2a). DU145 cells exhibited a higher level of transduction in comparison to TRAMP-C2 cells at each multiplicity of infection.

To quantify TRAMP-C2 and DU145 cells transduction with hAd-GFP at 10, 25, and 50 MOI, a flow cytometry analysis was performed 24 h post infection (Fig. 2b). An increase in the percentage of GFP positive cells was observed in both TRAMP-C2 and DU145 cells as the MOI was increased. However, when comparing similar treatments, TRAMP-C2 cells were significantly less affected than were DU145 as indicated by a GFP positive population of only 17.7 ± 2.8% at 50 MOI in TRAMP-C2 vs 59 ± 1.5% in DU145.

### TRAMP-C2 and DU145 cells express GFP following transduction with hAd-GFP

**Fig. 3:**
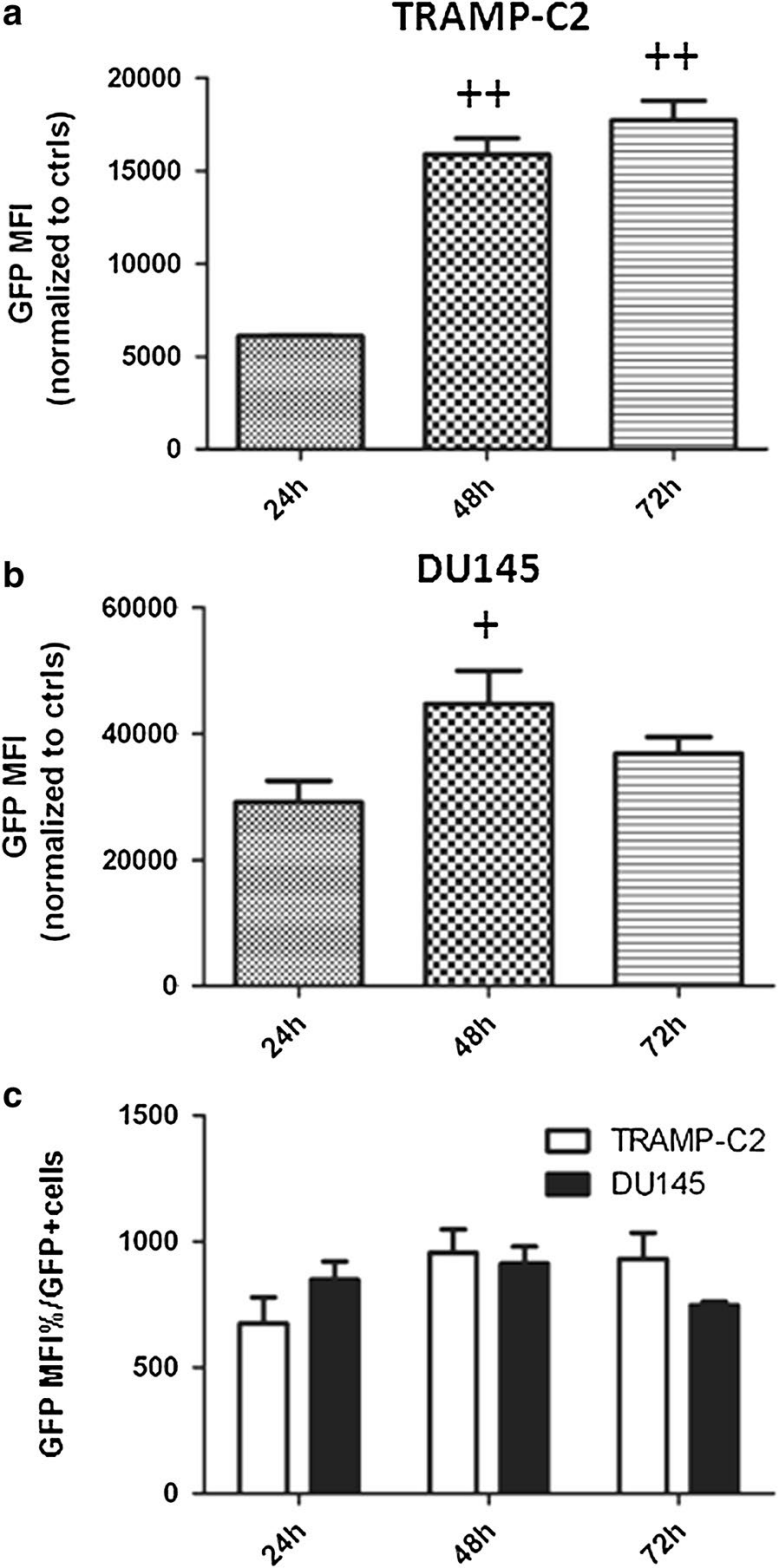
GFP protein expression in TRAMP-C2 and DU145. **a** TRAMP-C2 and **b** DU145 cells were transduced with 10 MOI of hAd-GFP and the median fluoresce intensity of GFP transgene (GFP MFI) was analyzed by flow cytometry at 24, 48 and 72 h post transduction. **c** GFP MFI was corrected by the percentage of GFP positive (GFP+) cells to compare GFP expression in equal number of transduced cells. Data are representative of three biological repeats analyzed by (**a**, **b**) one way ANOVA, Tukey’s post-test ^+^p<0.05, ^++^p < 0.01; **c** two ways ANOVA, Bonferroni’s post-test, *p < 0.05, **p < 0.01

The hAds-GFP used in this study express the GFP gene under the strong (CMV) cytomegalovirus constitutive promoter. In order to assess if mouse TRAMP-C2 and human DU145 prostate cancer cells were able to express GFP after transduction with hAd-GFP, both cell lines were infected with 10 MOI of hAds, and the median fluorescence intensity (MFI) was measured at 24, 48, and 72 h to determine the degree of GFP protein synthesis. We showed that the GFP transgene is expressed in both TRAMP-C2 (Fig. 3a) and DU145 (Fig. 3b) cell lines. When comparing human and murine cell lines, GFP MFI in DU145 was higher than TRAMP-C2 at all 3 times points corresponding to a greater transduction efficiency of hAds in human cells. However, when GFP MFI was corrected by the percentage of GFP^+^ cells detected at 24, 48 and 72 h, a similar GFP expression in both murine and human cells was observed (Fig. 3c).

### Microbubbles protect hAd-GFP from activation of the innate immune system

**Fig. 4:**
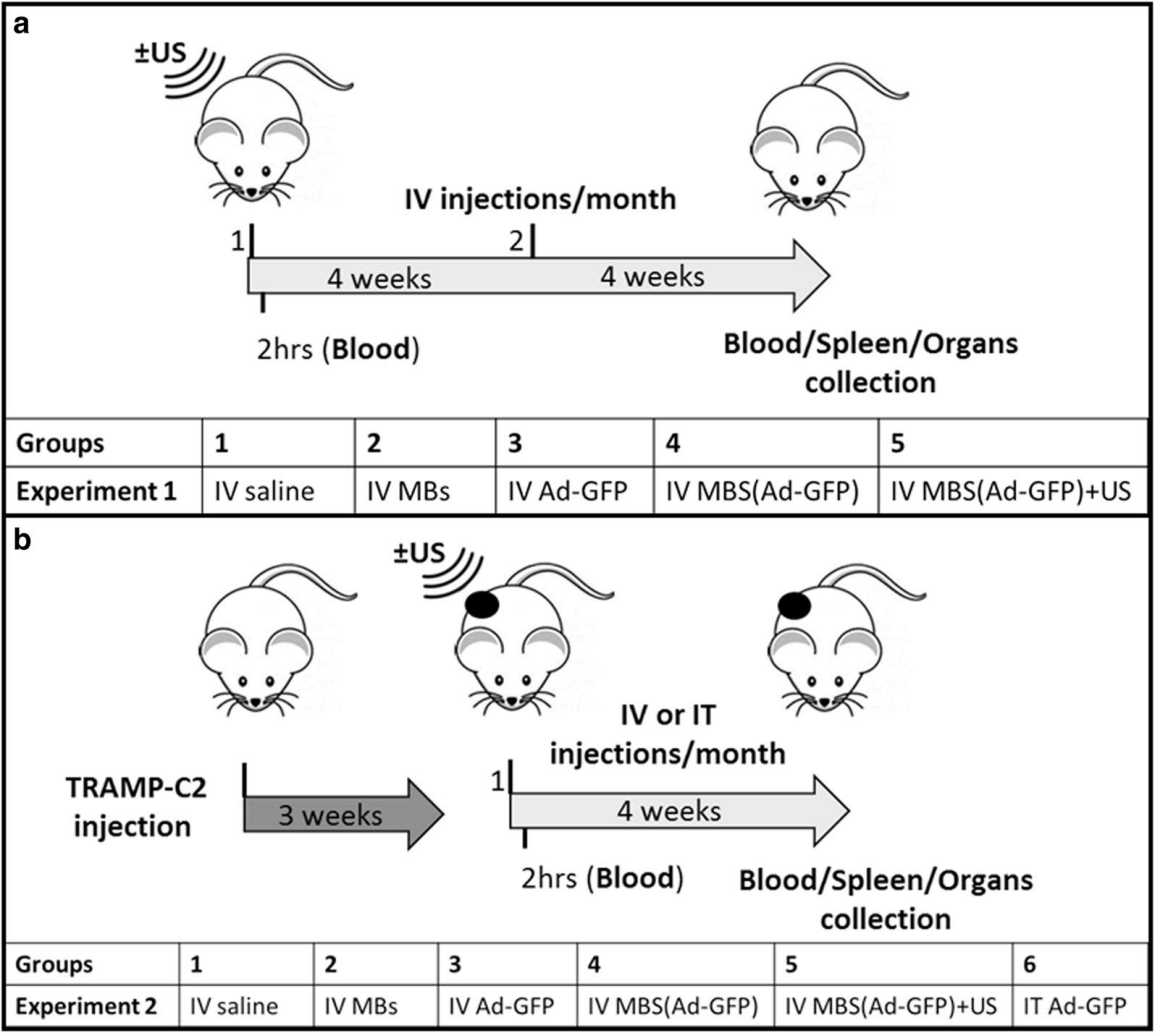
In vivo experimental design. Two in vivo experiments were performed. **a** In experiment 1 we used healthy C57BL/6. Mice were injected IV with either saline, MBs, hAd-GFP, MBs(hAd-GFP), or MBs(hAd-GFP) + US. Blood samples were collected 2 h post-injection to determine inflammatory cytokine response. After 4 weeks, mice were re-injected and 4 weeks later, mice were sacrificed and blood and organs were collected. **b** In experiment 2 we used C57BL/6 mice injected subcutaneously with 5 × 10^6^ TRAMP-C2 cells. When syngeneic tumor grafts were established (approximately 3 weeks post-injection of cancer cells, and with tumor volumes between 50 and 100 mm^3^) mice were injected IV with either saline, MBs, hAd-GFP, MBs(hAd-GFP), MBs(hAd-GFP) + US, or via IT with hAd-GFP. Two hours after the treatment, blood samples were collected to determine inflammatory cytokine response. At the endpoint of 4 weeks, mice were sacrificed and blood and organs were collected. Activation of the innate and adaptive immune response were evaluated in both experiments by ELISA (TNF-α, IL-6 and NAbs)

**Fig. 5:**
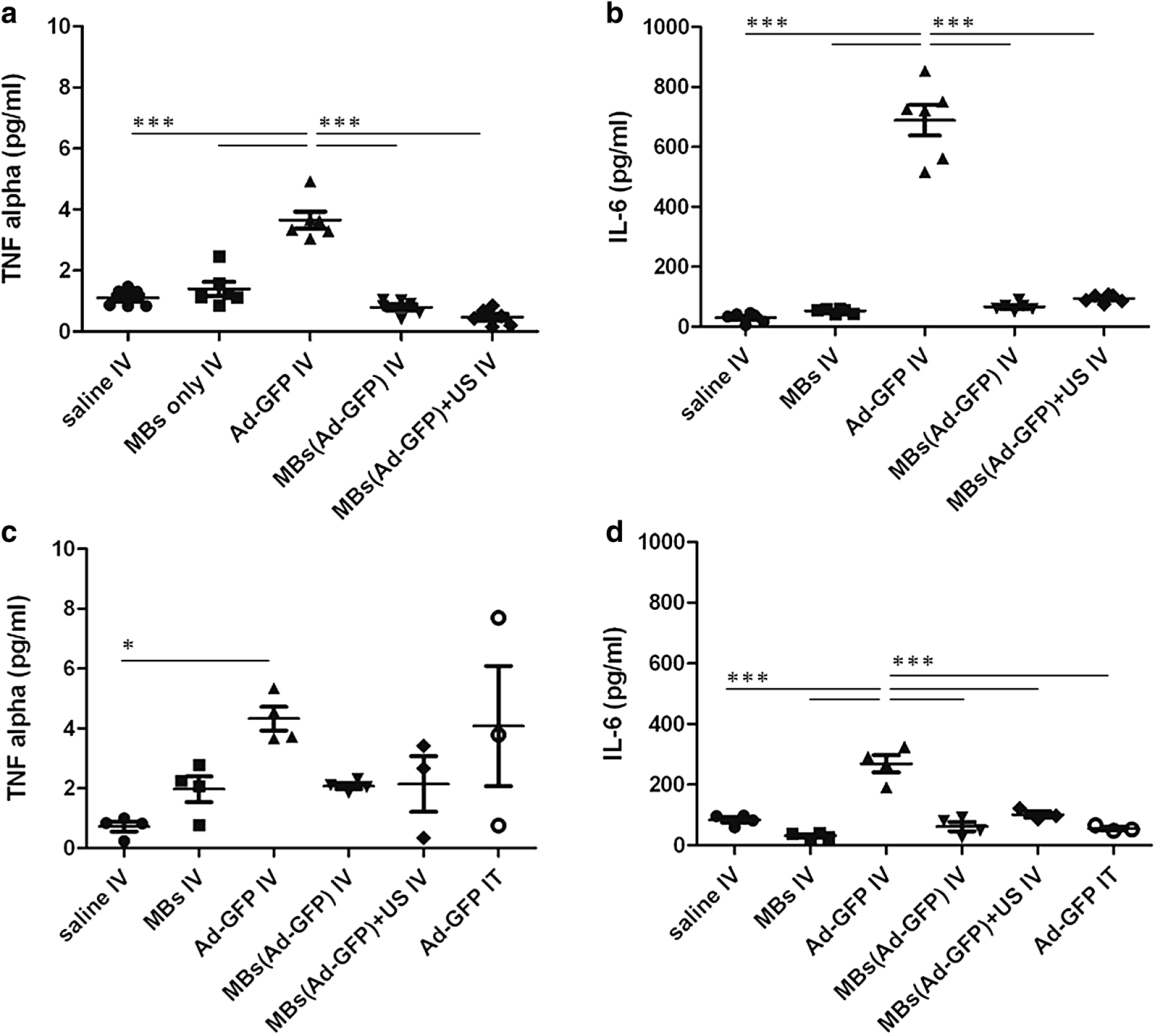
Quantification of serum inflammatory cytokines. Innate immunity was evaluated by sandwich ELISA by quantifying serum cytokines TNF alpha **a** EXP.1, and **c** EXP.2, and serum IL-6 **b** EXP.1, and **d** EXP.2. Groups means are represented as horizontal bars (EXP.1 n = 6 mice/group; XPp.2 n = 4 mice for saline IV, MBs IV, hAd-GFP IV, MBs(hAd-GFP) IV groups and n = 3 mice for MBs(hAd-GFP) + US IV and hAd-GFP IT. Data are analyzed by two way ANOVA and Tukey’s post-test, *p < 0.05, ***p < 0.001)

The first barrier to viral infection is the innate immunity, which is comprised of cellular and soluble components including complement, immunoglobulin, erythrocyte and clotting factor binding (depending on species), and inflammatory cytokines (TNF-α, IL-1β, IL-6) [13]. To evaluate the MBs’ ability to protect in immune competent animals the hAds from the innate immunity, the levels of serum inflammatory cytokines after IV and IT injection of both unprotected hAd-GFP and MB-protected hAd-GFP were measured using immunoassay solid-phase ELISAs. The experimental design is shown in Fig. 4. Direct IV injection of unprotected hAd-GFP in healthy C57BL/6 immunocompetent mice (Fig. 4a, EXP.1) induces a marked increase in both TNF-α (Fig. 5a) and IL-6 (Fig. 5b) in comparison to control groups (saline and MBs only). As anticipated, the administration of MB-protected hAd-GFP completely shielded the adenovirus, preventing the activation of innate immunity in both ultrasound-treated and non-treated groups. When C57BL/6 mice bearing a syngeneic tumor were used (Fig. 4b, EXP.2), we detected an increase in both TNF-α (Fig. 5c) and IL-6 (Fig. 5d) in hAd-GFP IV injected mice just as we did using C57BL/6 mice without tumor; however, the increase in IL-6 using hAd-GFP IV injected mice was less substantial than that of the C57BL/6 mice without tumor. When C57BL/6 mice bearing tumors received an IT injection, the level of TNF-α was again comparable to the hAd-GFP IV group, but IL-6 production was absent. As expected, we observed that the administration of hAd-GFP/MBs complexes allows for the evasion of adenovirus from the innate immunity with or without the use of ultrasound.

### Microbubbles protect hAd-GFP from the activation of the humoral response

**Fig. 6:**
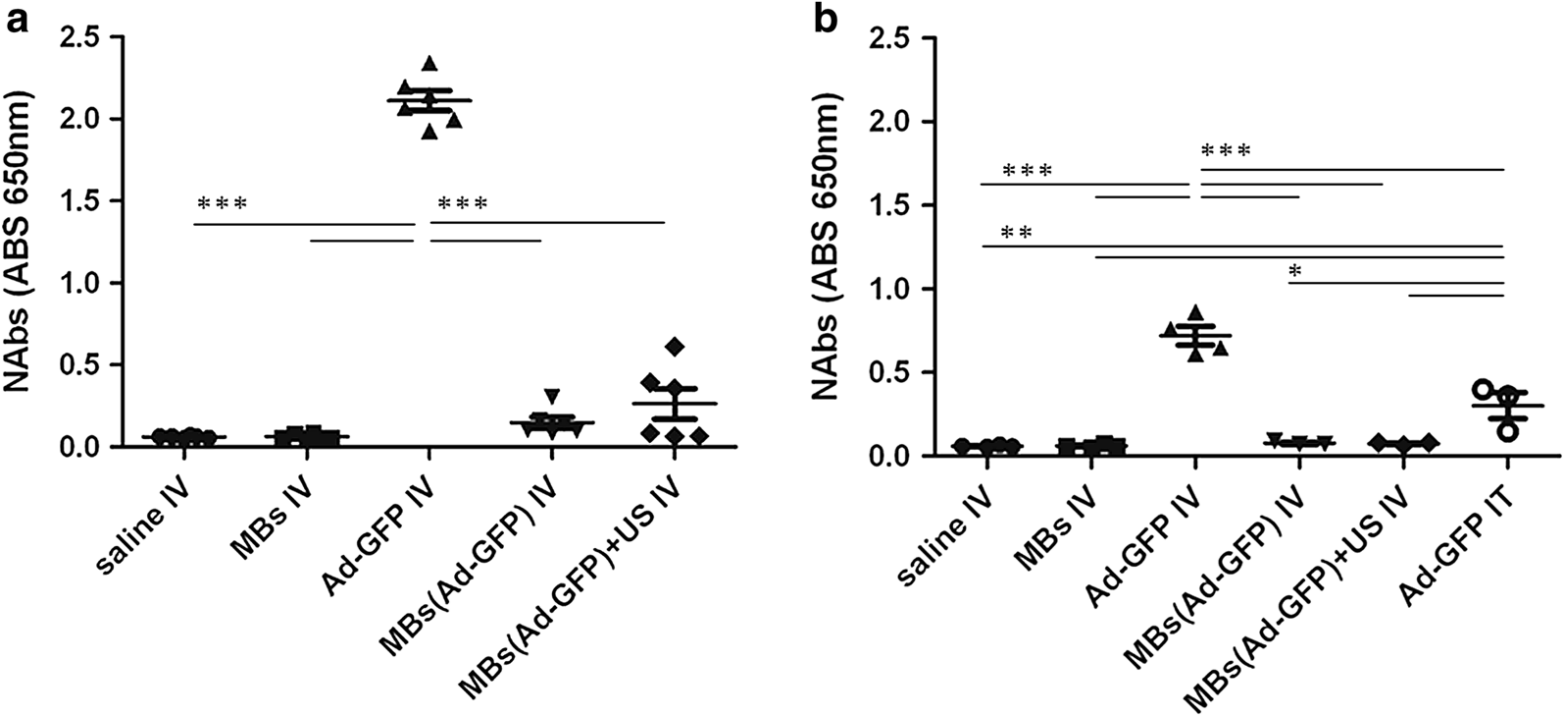
Detection of serum neutralizing antibodies. Humoral response was determined by direct ELISA of serum NAbs (IgG anti-hAd), **a** Exp.1, **b** Exp.2. Group’s means are represented as horizontal bars. (EXP.1 n = 6 mice/group; EXP.2 n = 4 mice for saline IV, MBs IV, hAd-GFP IV, MBs (hAd-GFP) IV groups and n = 3 mice for MBs(hAd-GFP) + US IV and hAd-GFP IT. Data are analyzed by two way ANOVA and Tukey’s post-test, *p < 0.05, **p < 0.01, ***p < 0.001)

The production of neutralizing antibodies specific for hAd-GFP (IgG anti-hAd) was evaluated using a direct ELISA assay. Blood was collected from the heart at the experimental endpoints of 2 months in C57BL/6 mice (Fig. 4a, EXP.1) and 1 month in C57BL/6 mice bearing a syngeneic tumor (Fig. 4b, EXP.2). There was a statistically higher titer of IgG anti-hAd when mice were injected intravenously with unprotected hAd-GFP. The production levels of neutralizing antibodies specific for hAd-GFP nearly doubled when mice received two IV injections of unprotected hAd-GFP in EXP.1 (Fig. 6a). C57BL/6 mice from EXP.2 (Fig. 6b) that received an IT injection of hAd-GFP showed a lower increase in NAbs when compared to the IV injection groups. Not surprisingly, we observed that the administration of MBs/hAd-GFP complexes ± US completely prevented the activation of a humoral response in immune competent mice.

### Microbubbles protect hAd-GFP from the activation of the cellular response

**Fig. 7:**
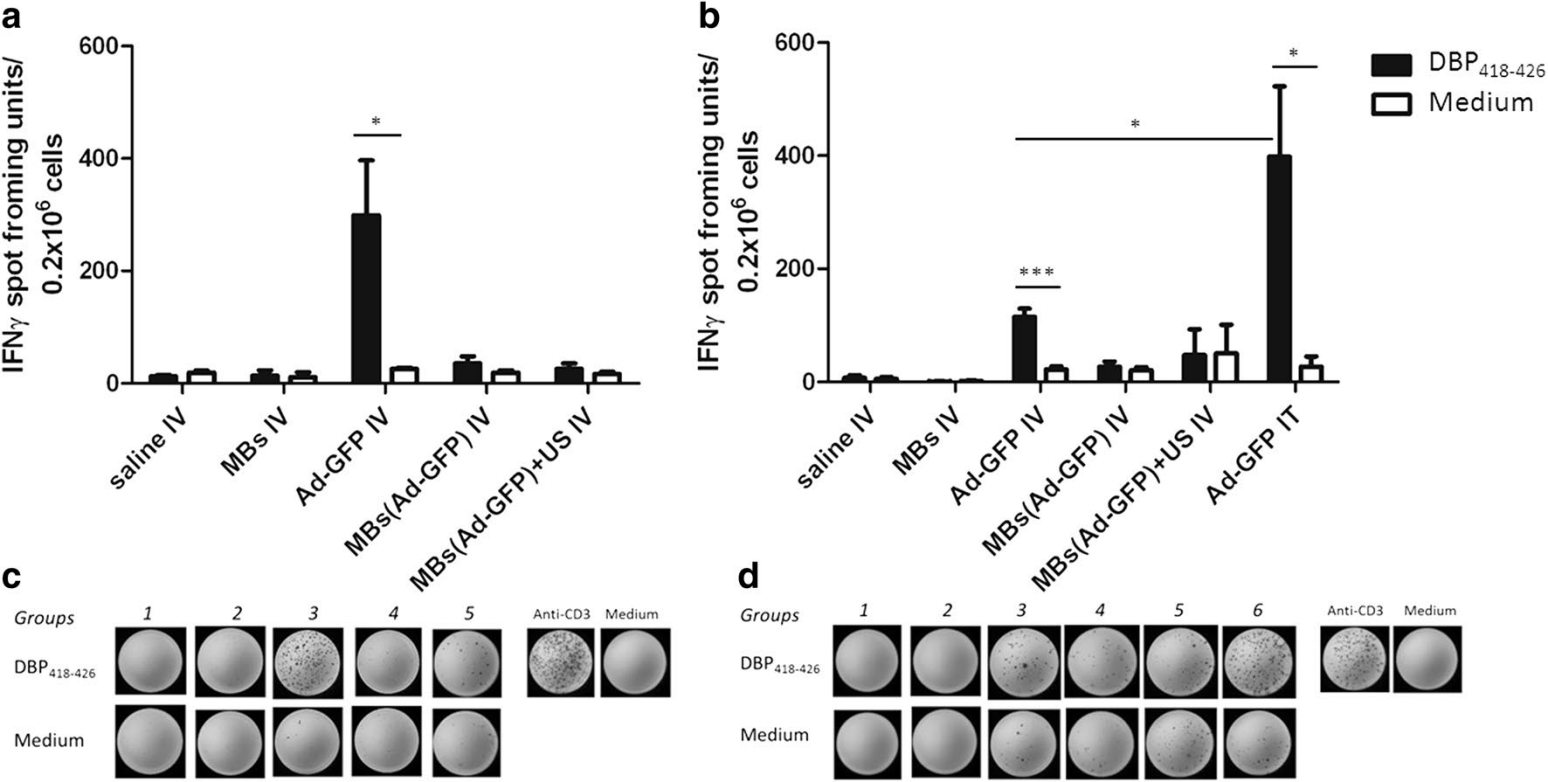
Detection of INF-γ producing CD8^+^ T. Cellular response was assay by ELIspot counting the number of INF-γ spots forming units after ex vivo stimulation of splenocytes with DBP_418–426_ peptide **a**, **c** EXP.1, **b**, **d** EXP.2. Group means are represented as in the histograms. **c**, **d** Exemplary samples. The nonspecific activator anti-CD3 antibody was used as positive control for the assay. (EXP.1 and EXP.2 n = 3 mice/group. Data are analyzed by multiple t-test of splenocytes from control and treated mice stimulated with media, spontaneous, or DBP_418–426_, stimulated, *p < 0.05, ***p < 0.001)

The presence of antigen-specific INF-γ producing CD8^+^ T cells was assessed by stimulating the splenocytes ex vivo with DBP_418–426_ peptide. The number of cells responding to the stimulation was evaluated using an ELISpot assay. Splenocytes were collected at the experimental endpoints. A statistically higher amount of INF-γ spot forming units were found in the stimulated splenocytes compared to the un-stimulated splenocytes in the IV-injected, unprotected hAd-GFP mice. The number of INF-γ spot forming units counted in samples from mice in EXP.1 (Fig. 7a, c) that received two IV injections of unprotected hAd-GFP was almost twofold of that which was observed in the mice from EXP.2 (Fig. 7b, d). Mice from EXP.2 (Fig. 7b) that received an IT injection of hAd-GFP showed a greater number of INF-γ spot forming units when compared to the IV injections. Notably, we did not observe a statistically significant activation of splenocytes after stimulation with DBP_418–426_ in mice that received MBs/hAd-GFP complexes ± US treatment indicating that the UMTD we developed prevented the activation of a humoral response in immune competent mice.

## Discussion

Prostate cancer is a very common cancer in men in the United States ranking as the third-leading cause of cancer death in men [31]. Primary prostate cancer can be treated successfully in many cases with surgical prostate resection, radiation, and hormonal therapy, with radiation therapy being used as the main choice for locally advanced prostate cancer. However despite receiving treatment, over a third of these patients will progress to an androgen-independent, radiation-resistant prostate cancer [32]. There is a need to develop more effective therapeutic approaches, and gene therapy represents a promising new treatment option. Therapeutic genes of choice include pro-apoptotic genes, tumor suppressor genes, antisense sequences for oncogenes, and anti-tumor DNA vaccines. Recent gene therapy clinical trials for prostate cancer have used Adenovirus as a highly efficient gene transducing tool [24]. Together with the Adeno-associated virus, adenoviral vectors belong to the category of the non-integrating vectors, they can be produced at a higher titer and display a robust expression of the therapeutic genes. These vectors can be easily engineered to make them safer and less immunogenic [33]. For example, the substitution of serotype 5 hexons with serotype 3 can protect adenovirus form inactivation by neutralizing antibody anti-hAd5 that are commonly circulating in patients due to preexisting immunization [34, 35]. In fact, the main challenges associated with the systemic delivery of adenoviral vector are not only the naïve immune response but also the immunity to the virus serotype stemming from natural infection and liver toxicity [13].

Adenoviruses interact with the host cell and internalize using specific receptors. Adenovirus 5 is one of the main serotypes currently used in the clinics, and it employs Coxsackie and Adenovirus Receptor (CAR) to adhere to target cells [36]. This receptor is expressed at low levels in primary tumors, including prostate cancer, when compared to established human cancer cell lines [37].

Infection and replication by human adenovirus has been thought to be restricted to human cells. Murine cells have been generally considered non-permissive, thereby limiting preclinical studies of gene transfer techniques [38]. However, here we showed that murine tissue could be transduced with hAds even if at a lower extent.

To overcome the aforementioned challenges through elicitation of the immune response and off-target viral distribution and expression, we have developed an image-guided gene transfer method (US Patent 8,454,937) utilizing a combination of lipid-encapsulated perfluorocarbon microbubbles (MBs) and ultrasonic waves (US) to enclose and protect hAds from the immune system to deliver the adenoviruses to a site-specific tissue bypassing the requirement of specific receptors [21, 25–27]. We have previously shown in immune compromised mice that this innovative gene transfer system can be used to specifically deliver hAd-GFP to a prostate tumor xenograft after systemic injection of the virus [25]. We demonstrated, by delivering a replication-deficient or a conditionally replication-competent adenovirus expressing the pro-apoptotic gene mda7/interleukin-24 enclosed in microbubble and in combination with ultrasound, that we could achieve sustained expression of the transgene in the sonoporated region and induce a reduction or complete eradication of a human prostate tumor xenograft [21]. Using this microbubble gene transfer method we were also able to radio-sensitize and reduce the tumor burden of a tumor xenograft of the prostate cell line DU145 by delivering replication-deficient human adenovirus expressing the tumor suppressor genes p53, and pRb [27]. Moreover, we confirmed our previously published data, showing that after reconstitution of microbubbles in the presence of adenovirus, the microbubbles need to be treated with human complement in order to inactivate any adenovirus loosely attached or included within the lipid shell and to achieve specific delivery of the hAds. The complement-treated microbubble-encapsulated adenovirus can be systemically injected intravenously into an immunocompetent C57BL/6 mouse without eliciting any innate immune response when compared to non-treated microbubbles or not protected adenovirus [24, 25].

The validity of our established image-guided gene therapy method has been confirmed by an independent laboratory that is using ultrasound-targeted microbubble (MB)-destruction to deliver conditionally replication-competent oncolytic adenoviruses that simultaneously produce a systemically active cancer-specific therapeutic cytokine [39] in prostate cancer.

The aim of the present study was to test if the microbubble/US system we have established [21, 24, 25, 27] could efficiently deliver human adenoviruses to a targeted diseased tissue protecting the virus from the innate and adaptive immunity using immunocompetent TRAMP-C2 mice (C57BL/6 background) as pre-clinical prostate cancer model.

Using an in vitro model, we compared the transduction efficiency of the hAd-GFP in the mouse TRAMP-C2 and human DU145 prostate cancer cell lines and found that the pattern of expression of the CAR receptors and integrins α_V_β_5_ and α_V_β_3_, all required for the adhesion and internalization of the adenovirus by the host cells, was comparable. However, notwithstanding this similarity, we observed a pronounced reduction in the uptake of the virus when comparing murine cells and human prostate cell line. This can be explained by the high sequence homology in the extracellular domain of CAR from human and mice [40, 41] and CAR-independent pathways for cell transduction [42]. However, we detected a dose-dependent increase of the GFP positive cells in both cell lines 24 h post-infection. Finally, and more relevant for the general purpose of our study, we showed that both mouse TRAMP-C2 and human DU145 prostate cancer cell lines were able to support an efficient expression of the GFP transgene regulated by the strong CMV promoter at 48 and 72 h post-transfection.

The second goal of this study was to test the ability of the microbubbles to protect the systemically delivered adenoviral vectors from the innate and adaptive immune system of an immunocompetent mouse in vivo, using our image-guided delivery system. For this purpose, we used the TRAMP-C2 model of prostate cancer. From the original TRAMP mouse, that spontaneously develops prostate cancer, several cell lines have been established, such as TRAMP-C1 and C2, and these can be used to establish syngeneic subcutaneous grafts in C57BL/6 mice [43]. In our study, we used healthy immunocompetent C57BL/6 mice and mice bearing a syngeneic TRAMP-C2 prostate tumor to better represent the immune response of cancer patients.

Adenoviruses are able to induce a strong inflammatory response, which at its first step involves the activation of NK, professional APC, neutrophils, the complement cascade and the secretion of cytokines [13]. Dendritic cells in the spleen have been demonstrated to be directly transduced by systemically administered adenovirus resulting in the induction of IL-6, IL-12, and other cytokines [44]. The administration of human adenoviral vectors in protocols of gene therapy can lead to side effects in patients such as liver toxicity, thrombocytopenia and acute inflammation [45]. In order to assess if the microbubbles could protect the hAds from the activation of the innate immunity following systemic delivery, we injected the hAds enclosed in microbubbles through the tail vein of the mice. Two hours after intravenous injection, corresponding to the previously reported median time of secretion peak for TNF-α and IL-6 in C57BL/6 mice [46–48], blood samples were collected from the treated mice and levels of cytokines in their serum were quantified. We observed that the microbubbles completely protected hAds from eliciting an immune response, as showed by the absence of inflammatory cytokines when compared to the expected and well-documented response obtained after injection of non-protected hAds [11, 13, 44, 49, 50]. We did not observe any difference among ultrasound-treated and non-treated mice (EXP.1 to the right kidney, EXP.2 to the tumor on the right flank), confirming the high stability of the microbubbles used. Furthermore, we noticed a very low level or absence of virus leak when bubbles cavitation was induced by sonoporation. Mice that received an intratumoral injection of the hAds showed an increase of only TNF-α expression, which naturally precedes IL-6 [46, 48], suggesting that anatomical barriers such as the tight junction between tumor cells may have reduced the path of the viral vector delaying the elicitation of innate immunity.

The second barrier to viral infection is the adaptive immunity, which is comprised of activated CD4^+^, CD8^+^ T cells and antibody-secreting plasma cells [13]. During a scheduled treatment of cancer gene therapy, the real obstacle to effectively repeating systemic administrations of replication-deficient adenoviral vectors is the inactivation of the virus by complement proteins, pre-existing anti-viral and neutralizing antibodies that can reduce the efficiency of transfection [11, 13]. In order to assess the activation of the adaptive immunity, at the experimental end point of 1 month from the last injection of microbubble encapsulated hAds either in combination with US or not, we measured the levels of serum IgG anti-adenovirus. We observed that the microbubbles completely protected hAds injected intravenously from eliciting a humoral response as shown by the absence of a statistically significant increase in secretion of anti-hAds antibodies. We could not detect differences among mice that received ultrasound treatment or not, confirming once again our previous observations. On the other hand, we detected a robust production of neutralizing antibodies in mice injected intravenously with unprotected hAds as observed by others [11, 13]. Additionally, in mice injected intravenously with unprotected hAds, the relative number of neutralizing antibodies detected was twice as much in EXP.1 compared to EXP.2 due to the treatment schedule. Instead, intratumoral injection of hAds induced a lower titer of neutralizing antibody probably due to the target tissue characteristics and the route of administration.

To assess the activation of cell-mediated immunity, we investigated the incidence of antigen specific INF-γ producing CD8^+^ T cells. The vector we used in our experiments is a 1st generation, E1 deleted adenovirus that still allows for the leaky late gene expression of some viral products including the nonstructural DNA Binding Protein (DBP). DBP contains a MHC-class I restricted epitope that have been shown to be a CD8^+^ principal epitope in C57BL/6 mice [30]. In order to detect the presence of DBP specific INF-γ producing CD8^+^ T cells, we performed an INF-γ ELIspot assay. We collected splenocytes at the experimental end points and stimulated them ex vivo with the DBP peptide and INF-γ spot forming units were counted. We observed that the systemic injection of microbubble encapsulated hAd-GFP with or without the use of US treatment does not induce a statistically significant increase in the number of spots observed when compared to the spontaneous release of INF-γ in both healthy mice (EXP.1) and mice bearing a prostate tumor (EXP.2). Conversely, we observed a strong activation of CD8^+^ T cells in the positive controls (IV and IT injections of naked hAds) and the highest count of INF-γ spot forming units was detected after intratumoral injection of hAd-GFP confirming that the route of administration and viral dose administered may affect the type of immune response [30, 51, 52].

## Conclusions

These results demonstrated that our MBs/US-guided gene delivery system can effectively protect a viral vector from the activation of both humoral and cellular immunity response. Also, our data provides evidence that the TRAMP-C2 mouse model of prostate cancer is a suitable system to study the feasibility of this novel image-guided gene transfer technique in immune competent animals offering the milestone and opportunity to further translate this ultrasound-mediated MBs/hAd delivery system from the bench to the bedside.

## Additional file

**Additional file 1: Figure S1.** Virus Titration. Virus stocks used for the *in vivo* experiments were tittered infecting HEK-293 cells at six serial dilutions (10^−2^, 10^−3^, 10^−4^, 10^−5^, 10^−6^, and 10^−7^) of Ad-GFP. The TCID_50_ method was used to calculate the titer. Representative pictures are showed for each dilution tested and the control (only media). **Figure S2.** Enhancement of Human Adenoviral transduction efficiency in TRAMP-C2 and DU145 following US-mediated MBs(Ad) delivery system. TRAMP-C2 and DU145 cells were transduced with 10MOI of Ad-GFP or MBs(Ad-GFP)+US. Cells receiving MBs(Ad-GFP) were treated with US for 1 minute. 24 hours after infection, the percentage of cells transduced were determined by Flow Cytometry. Data are representative of three biological repeats, analyzed by Student T-test (Ad-GFP vs. MBs(Ad-GFP)+US). *p < 0.05, **p < 0.001.

## Abbreviations

APC: antigen presenting cell
CAR: Coxsackie and Adenovirus Receptor
CMV: cytomegalovirus
CTL: cytotoxic T lymphocyte
DBP: DNA binding protein
FBS: fetal bovine serum
GFP: green fluorescent protein
hAds: human adenovirus
IL-6: interleukin 6
IT: intratumoral
IV: intravenous
MBs: microbubbles
MFI: median fluorescence intensity
MOI: multiplicity of infection
NAbs: neutralizing antibodies
NK: natural killer
TNF-α: tumor necrosis factor α
US: ultrasound
UTMD: ultrasound targeted microbubbles destruction
