# Supplementary material for: Microbubble-mediated delivery of human adenoviruses does not elicit innate and adaptive immunity response in an immunocompetent mouse model of prostate cancer
Source: J Transl Med. 2019 Jan 11;17:19. doi: 10.1186/s12967-019-1771-0 (PMC6329087; doi:10.1186/s12967-019-1771-0)
Supplement: Supplementary file 1 — Additional file 1: Figure S1. Virus Titration. Virus stocks used for the in vivo experiments were tittered infecting HEK-293 cells at six serial dilutions (10−2, 10−3, 10−4, 10−5, 10−6, and 10−7) of Ad-GFP. The TCID50 method was used to calculate the titer. Representative pictures are showed for each dilution tested and the control (only media). Figure S2. Enhancement of Human Adenoviral transduction efficiency in TRAMP-C2 and DU145 following US-mediated MBs(Ad) delivery system. TRAMP-C2 and DU145 cells were transduced with 10MOI of Ad-GFP or MBs(Ad-GFP)+US. Cells receiving MBs(Ad-GFP) were treated with US for 1 minute. 24 hours after infection, the percentage of cells transduced were determined by Flow Cytometry. Data are representative of three biological repeats, analyzed by Student T-test (Ad-GFP vs. MBs(Ad-GFP)+US). *p < 0.05, **p < 0.001. [file 12967_2019_1771_MOESM1_ESM.docx]

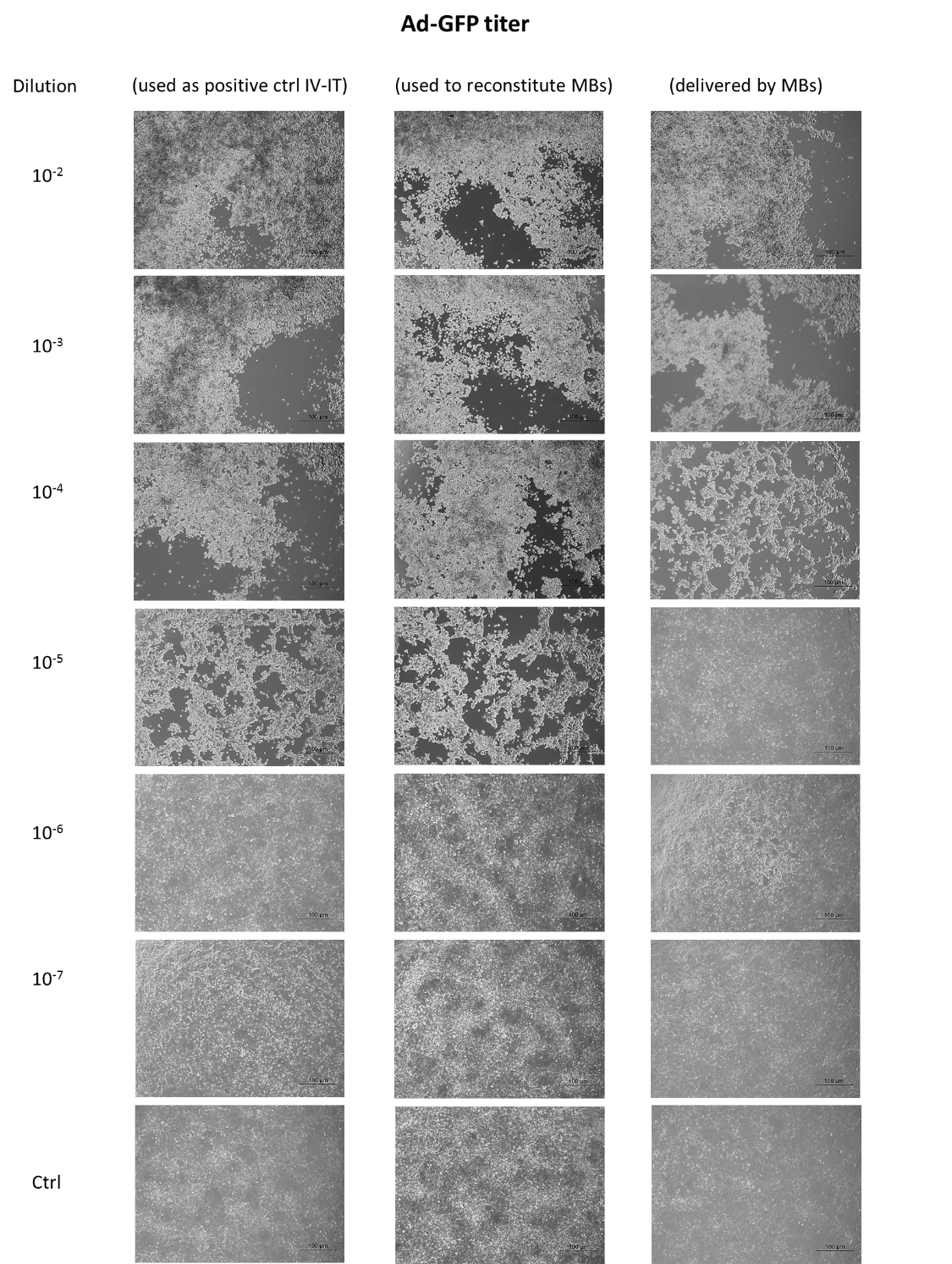


**Additional file 1: Figure S1. Virus Titration**. Virus stocks used for the *in vivo* experiments were tittered infecting HEK-293 cells at six serial dilutions (10^-2^, 10^-3^, 10^-4^, 10^-5^, 10^-6^, and 10^-7^) of Ad-GFP. The TCID_50_ method was used to calculate the titer. Representative pictures are showed for each dilution tested and the control (only media).


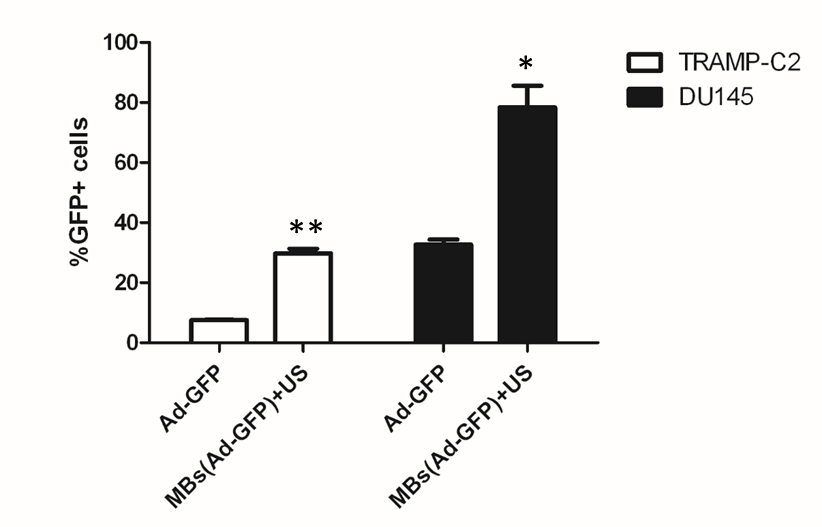


**Additional file 1: Figure S2. Enhancement of Human Adenoviral transduction efficiency in TRAMP-C2 and DU145 following US-mediated MBs(Ad) delivery system.** TRAMP-C2 and DU145 cells were transduced with 10MOI of Ad-GFP or MBs(Ad-GFP)+US. Cells receiving MBs(Ad-GFP) were treated with US for 1 minute. 24 hours after infection, the percentage of cells transduced were determined by Flow Cytometry. Data are representative of three biological repeats, analyzed by Student T-test (Ad-GFP vs. MBs(Ad-GFP)+US).

* p<0.05, **p<0.001.
